# Supplementary material for: TRPS1 maintains luminal progenitors in the mammary gland by repressing SRF/MRTF activity
Source: Breast Cancer Res. 2024 May 3;26:74. doi: 10.1186/s13058-024-01824-7 (PMC11067134; doi:10.1186/s13058-024-01824-7)

Uncropped immunoblots for Fig. 1b

Blot 1: TRPS1

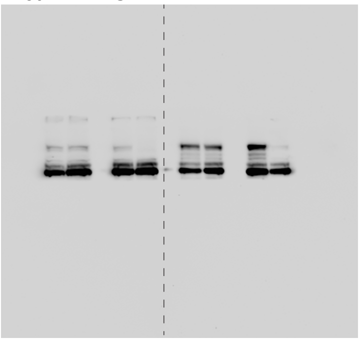

Blot 2:TRPS1
(long exposure of
Blot 1 cut at dashed line)

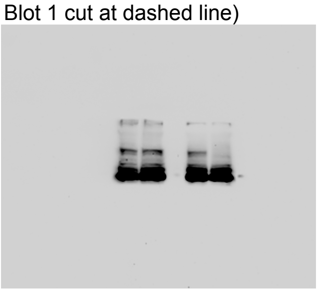

Blot 3: VINCULIN

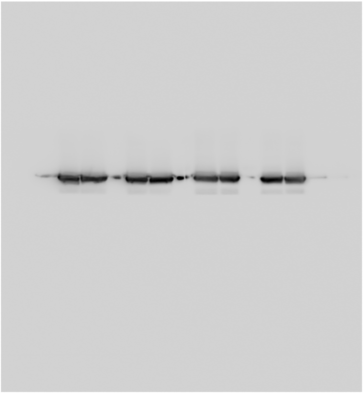

Supplement: Supplementary file 1 — Additional file 1. Additional methods. [file 13058_2024_1824_MOESM1_ESM.pdf]
