# Supplementary material for: TRPS1 maintains luminal progenitors in the mammary gland by repressing SRF/MRTF activity
Source: Breast Cancer Res. 2024 May 3;26:74. doi: 10.1186/s13058-024-01824-7 (PMC11067134; doi:10.1186/s13058-024-01824-7)
Supplement: Supplementary file 6 — Additional file 6 :Reagents list as pdf. [file 13058_2024_1824_MOESM6_ESM.pdf]

Additional Table 5: Reagents

REAGENT	Source	Identifier	Concentration
**Antibodies**			
Biotin anti-TER-119, Clone:TER-119	ThermoFisher Scientific	13-5921-82	1:50
Biotin anti-CD31, Clone:390	ThermoFisher Scientific	13-0311-82	1: 50
Biotin anti-CD45, Clone:30-F11	ThermoFisher Scientific	13-0451-82	1:50
PE-anti mouse EpCAM, Clone:1B7	Biolegend	12-9326-42	1: 100
APC/Fire™750 anti-human/mouse CD49f antibody, Clone: 93	Biolegend	313631	1:100
APC anti-mouse CD117 (c-kit)-Clone:2B8	Biolegend	105812	1: 100
Streptavidin-eFluor450	ThermoFisher Scientific	48-4317-82	1:200
PE-Vio 770 Anti-mouse CD14	Miltenyi	130-115-560	1:100
TotalSeq™-A0449 anti-mouse CD326 (Ep-CAM) Antibody	Biolegend	118237	0,5 µg
TotalSeq™-A0070 anti-human/mouse CD49f Antibody:	Biolegend	313633	0,5 µg
TotalSeq™-A0238 Rat IgG 2α, λ Isotype control	Biolegend	400571	0,5 µg
Anti-TRPS1	Abcam	ab209664	1:200
Anti-mouse Krt8	biolegend	904804	1:500
Anti-mouse Krt8	Developmental Studies Hybridoma Bank	TROMA-I-s	2 ug/mL
Anti-tGFP	OriGene	TA150041	1:400
AF488 Anti-mouse IgG	ThermoFisher Scientific		1:400
AF546 anti-Rabbit IgG	ThermoFisher Scientific		1:400
AF647 anti-Rat IgG	ThermoFisher Scientific		1:400
Anti-Vinculin (hVIN-1)	Sigma-Aldrich	#V9131	1:10000
Anti-SRF (D71A9) XP®	Cell signaling	5147T	1:100
Anti-mouse IgG-HRP	Santa Cruz	sc-2314	1:5000

Anti-rabbit IgG-HRP	Santa Cruz	sc-2313	1:5000
**Chemicals, Peptides, and Recombinant Proteins**			
Gibco Fetal Bovine Serum	ThermoFisher Scientific	F7524-500ML	
Leibovitz's L-15 Medium (500 mL)	ThermoFisher Scientific	11415064	
DMEM F-12 Glutamax	ThermoFisher Scientific	12634028	
B-27® Supplement (50X)	ThermoFisher Scientific	17504044	
N-2 Supplement (100X)	ThermoFisher Scientific	17502048	
Murine Noggin, 100µg	Peprotech	250-38	
Recombinant Mouse Neuregulin-1/NRG1 Protein, CF, 50µg	Peprotech	9875-NR-050	
Leibovitz's L-15 Medium (500 mL)	ThermoFisher Scientific	11415064	
Corning® Matrigel® Growth Factor Reduced (GFR) Basement Membrane Matrix, *LDEV-free,	Corning	354230	
TrypLE™ Express Enzyme (1X)	ThermoFisher Scientific	12604013	
Cultrex Organoid Harvesting Solution	R&D systems	3700-100-01	
RPMI 1640 (+GlutaMax)	ThermoFisher Scientific	21875091	
EpiCult-B Mouse Medium Kit	STEMCELL Technologies	5610	
SYTOX Blue dead cell stain	ThermoFisher Scientific	S34857	
Giemsa stain	Sigma	GS500	
**Critical Commercial Assays**			
innuMIX qPCR DSGreen Standard	Analytik Jena	845-AS-1300200	
RNeasy® Micro kit	Qiagen	74004	
NEBNext® Ultra RNA Library Prep kit for Illumina	NEB	E7530L	
Chromium Single Cell 3′ Library Construction Kit v3	10x Genomics	1000078	
Chromium Next GEM Single Cell ATAC Library & Gel Bead Kit v1.1	10x Genomics	1000176	
MinElute PCR Purification Kit	Qiagen	28006	

Agencourt AMPure XP	Beckman Coulter	A63881	
NEBNext® High-Fidelity 2X PCR Master Mix	NEB	M0541L	
Passive Lysis Buffer	Promega	E194A	
Gentle Collagenase/Hyaluronidase 10X	STEMCELL Technologies	7919	
