# Supplementary material for: TRPS1 maintains luminal progenitors in the mammary gland by repressing SRF/MRTF activity
Source: Breast Cancer Res. 2024 May 3;26:74. doi: 10.1186/s13058-024-01824-7 (PMC11067134; doi:10.1186/s13058-024-01824-7)
Supplement: Supplementary file 7 — Additional file 7 : Oligonucleotides list as pdf. [file 13058_2024_1824_MOESM7_ESM.pdf]

Additional Table 6: Oligonucleotides

Primer / oligo	Sequence
**Sc-Seq**	
RPI1	CAAGCAGAAGACGGCATACGAGATCGTGATGTGACTGGAGTTCCTTGGCACCCGAGAATTCCA
RPI2	CAAGCAGAAGACGGCATACGAGATACATCGGTGACTGGAGTTCCTTGGCACCCGAGAATTCCA
10X Genomics SI	AATGATACGGCGACCACCGAGATCTACACTCTTTCCCTACACGACGCTC
**Genotyping**	
**shRNA genotyping**	
shTrps1#1_Fw	AAGCCACAGATGTATCTTCTAATAA
shTrps1#2_Fw	AAGCCACAGATGTATTCGTATTTAC
shRen_Fw	AAGCCACAGATGTATAGATAAGCAT
Col1a1_Rev45	CACCCTGAAAACTTTGCCCC
**Col1a1 genotyping**	
Col1a1_Fw	AATCATCCCAGGTGCACAGCATTGCG
Col1a1_Rv	CTTTGAGGGCTCATGAACCTCCCAGG
SAdpA_Rv2	AAGACCGCGAAGAGTTTGTC
**CAG-lsl-RIK genotyping**	
rtTA_Rv2	CGCTTGTTCTTCACGTGCGA
rtTA_Fw (lsl)	AAAAACTCCCACACCTCCC
**Rosa26 genotyping**	
Rosa_D	TCAGTAAGGGAGCTGCAGTGG
Rosa_B	GCGAAGAGTTTGTCCTCAACC
Rosa_C	GGAGCGGGAGAAATGGATATG
**K8-CreER genotyping**	
oIMR7338	CTAGGCCACAGAATTGAAAGATCT
oIMR7339	GTAGGTGGAAATTCTAGCATCATCC
oIMR1084	GCGGTCTGGCAGTAAAAACTATC
oIMR1085	GTGAAACAGCATTGCTGTCACTT
**CAG-rtTA3 genotyping**	
SApA_For1	CTGCTGTCCATTCCTTATTC
CH8_Rev2	CGAAACTCTGGTTGACATG
CH8_For1	TGCCTATCATGTTGTCAAA
**qRT-PCR**	
mTrps1_Fw	GGTACAGAGGCCACCAGTTAT
mTrps1_Rev	GGCTCTCCTTCTACACTTTTGG
mAreg_Fw	GGTCTTAGGCTCAGGCCATTA
mAreg_Rev	CGCTTATGGTGGAAACCTCTC
mCald1_Fw	ATGGTAGAGGAGAAAACACCAGA
mCald1_Rev	CCATCCCCTTCTATTTTGGACTC
mb2M_1_Fw	AGCCGAACATACTGAACTGCTACG
mb2M_1_Rev	CGGCCATACTGTCATGCTTAACTC
mActa2_Fw	GTCCCAGACATCAGGGAGTAA
mActa2_Rev	TCGGATACTTCAGCGTCAGGA
mMyl9_Fw	ACAGCGCCGAGGACTTTTC
mMyl9_Rev	AGACATTGGACGTAGCCCTCT
mcKit_Fw	GCCTGACGTGCATTGATCC
mcKit_Rev	AGTGGCCTCGGCTTTTTCC
mElf5_Fw	ATGTTGGACTCCGTAACCCAT
mElf5_Rev	GCAGGGTAGTAGTCTTCATTGCT
mCd14_Fw	ACTTCTCAGATCCGAAGCCAG
mCd14_Rev	CCGCCGTACAATTCCACAT
mCtgf_Fw	CAGCATGGACGTTCGTCTG
mCtgf_Rev	AACCACGGTTTGGTCCTTGG
**shRNAs**	
shRen	AAGGTATATTGCTGTTGACAGTGAGCGCAGGAATTATAATGCTTATCTATAGTGAAGCCACA GATGTATAGATAAGCATTATAATTCCTATGCCTACTGCCTCGGACTTCAAGGGGCTA
shTrps1#1	AAGGTATATTGCTGTTGACAGTGAGCGAGGCGAGCAGATTATTAGAAGATAGTGAAGCCACA GATGTATCTTCTAATAATCTGCTCGCCGTGCCTACTGCCTCGGACTTCAAGGGGCTA
shTrps1#2	AAGGTATATTGCTGTTGACAGTGAGCGCCCGAGCCTGAGTAAATACGAATAGTGAAGCCACA GATGTATTCGTATTTACTCAGGCTCGGATGCCTACTGCCTCGGACTTCAAGGGGCTA
