# Supplementary material for: TRPS1 maintains luminal progenitors in the mammary gland by repressing SRF/MRTF activity
Source: Breast Cancer Res. 2024 May 3;26:74. doi: 10.1186/s13058-024-01824-7 (PMC11067134; doi:10.1186/s13058-024-01824-7)
Supplement: Supplementary file 8 — Additional file 8: Fig. S1: Breeding schemes, Fig. S2: Mouse phenotypic analysis timeline table, Fig. S3: additional UMAP plot of CITE-Seq data. Figs. S4-S9 and S11: cell sorting strategies employed for the different experiments.Fig. S10: sc-ATAC-Seq experiments showing that TRPS1 prevents LP differentiation Fig. S12: CFC assay showing the effect of the Y-27632 ROCK inhibitor on the colony forming ability of shRNA-expressing Luminal progenitors isolated from untreated shRen and shTrps1 mice. [file 13058_2024_1824_MOESM8_ESM.pdf]

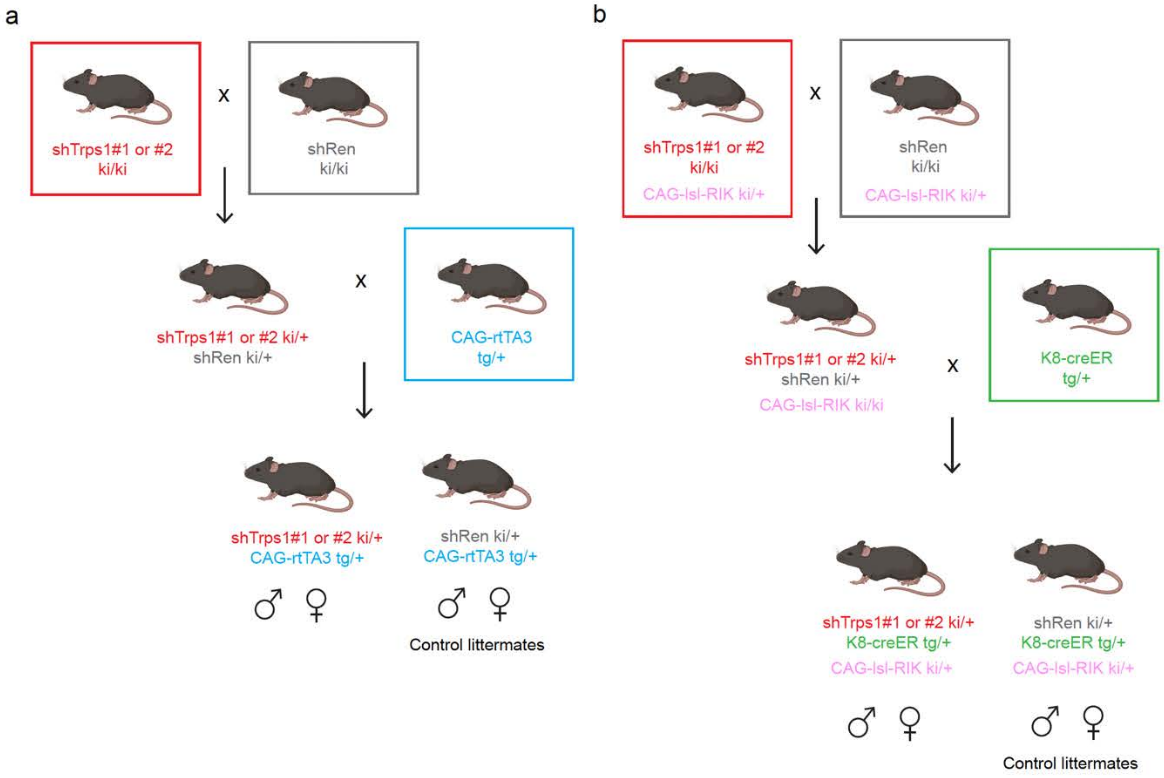


**Additional Figure 1: Breeding Schemes**

(a) generation of animals ubiquitously expressing shTrps1: homozygous shTrps1 (ki/ki) are crossed with homozygous shRen (ki/ki) to obtain progeny carrying both shTrps1 (ki/+) and shRen (ki/+) alleles. These animals are then crossed with CAG-rtTA3 heterologous (tg/+) animals to obtain shTrps1 ki/+, CAG-rtTA3 tg/+ experimental animals and shRen ki/+ CAG-rtTA3 tg/+ control littermates.

(b) generation of animals expressing shTrps1 specifically in the luminal compartment: animals homozygous for shTrps1 (ki/ki) and carrying a CAG-lsl-RIK allele (ki/+) are crossed with animals homozygous for shRen (ki/ki) and carrying a CAG-lsl-RIK allele (ki/+) to obtain progeny carrying both shTrps1 (ki/+) and shRen (ki/+) alleles and homozygous for CAG-lsl-RIK (ki/ki). These animals are then crossed with K8-CreER (tg/+) animals to obtain shTrps1 ki/+, CAG-lsl-RIK ki/+, K8-CreER tg/+ experimental animals and shRen ki/+, CAG-rtTA3 tg/+, K8-CreER tg/+ control animals.

| Screen | Methods |
|---|---|
| Behavior | Open field test |
| Neurology | Modified SHIRPA, grip strength |
| Behavior | Accoustic startle response (ASR), PPI |
| Neurology | Rotarod |
| Clinical chemistry | After fasting |
| Nociception | Hot plate |
| Dysmorphology | Anatomical observation |
| Allergy | Transepidermal water loss (TEWL), body surface temperature |
| Energy metabolism | Indirect calorimetry, NMR |
| Clinical chemistry | IpGTT |
| Cardiology | Awake ECG, Echocardiography |
| Eye | OCT, LIB, drum |
| Neurology | Auditory brain stem response (ABR) |
| Dysmorphology | X-ray, DEXA |
| Energy metabolism | NMR |
| Clinical chemistry | Clinical chemical analysis, hematology |
| Immunology | FACS, Immunoglobulin isotyping |
| Allergy | MSD Panel 3, Lymphocyte proliferation assay |
| Pathology | Macro & microscopic analysis |

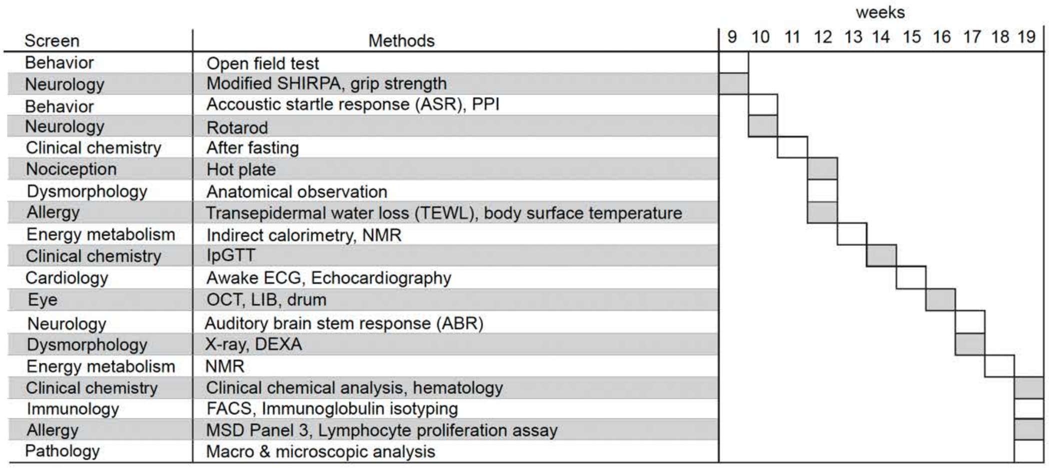


## Additional Figure 2: Mouse Phenotypic analysis timeline

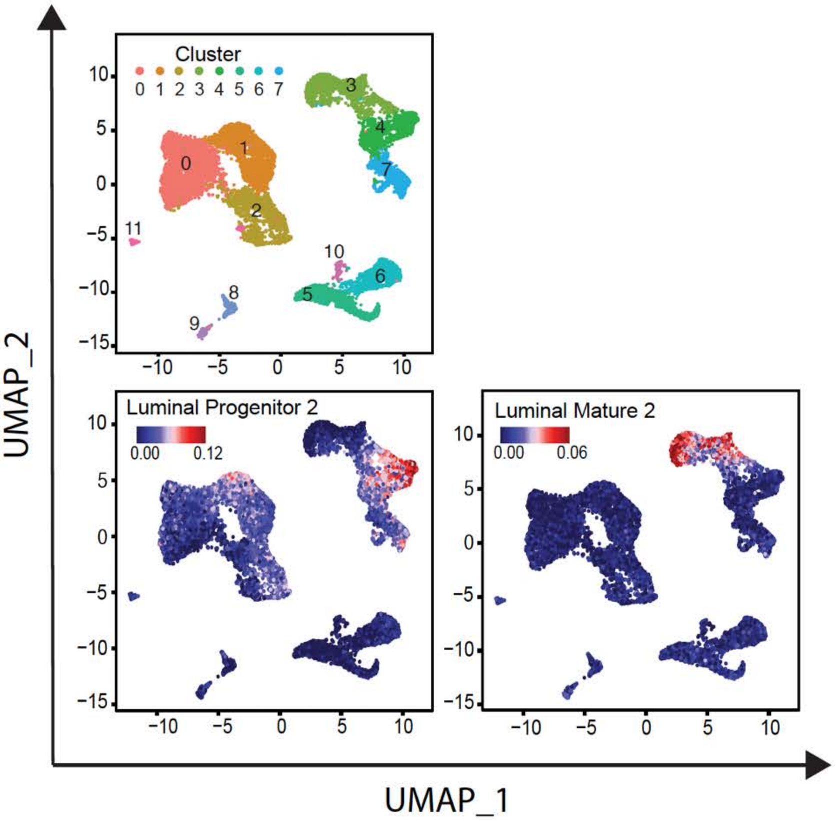


**Addtional Figure 3:**
UMAP dimensionality reduction plots of the CITE-Seq data from shTrps1 and shRen mammary gland cells combined (see Figure 3) showing the gene set activity based on AUCs of a luminal progenitor and a luminal mature gene set published by Lim et al. (2010).

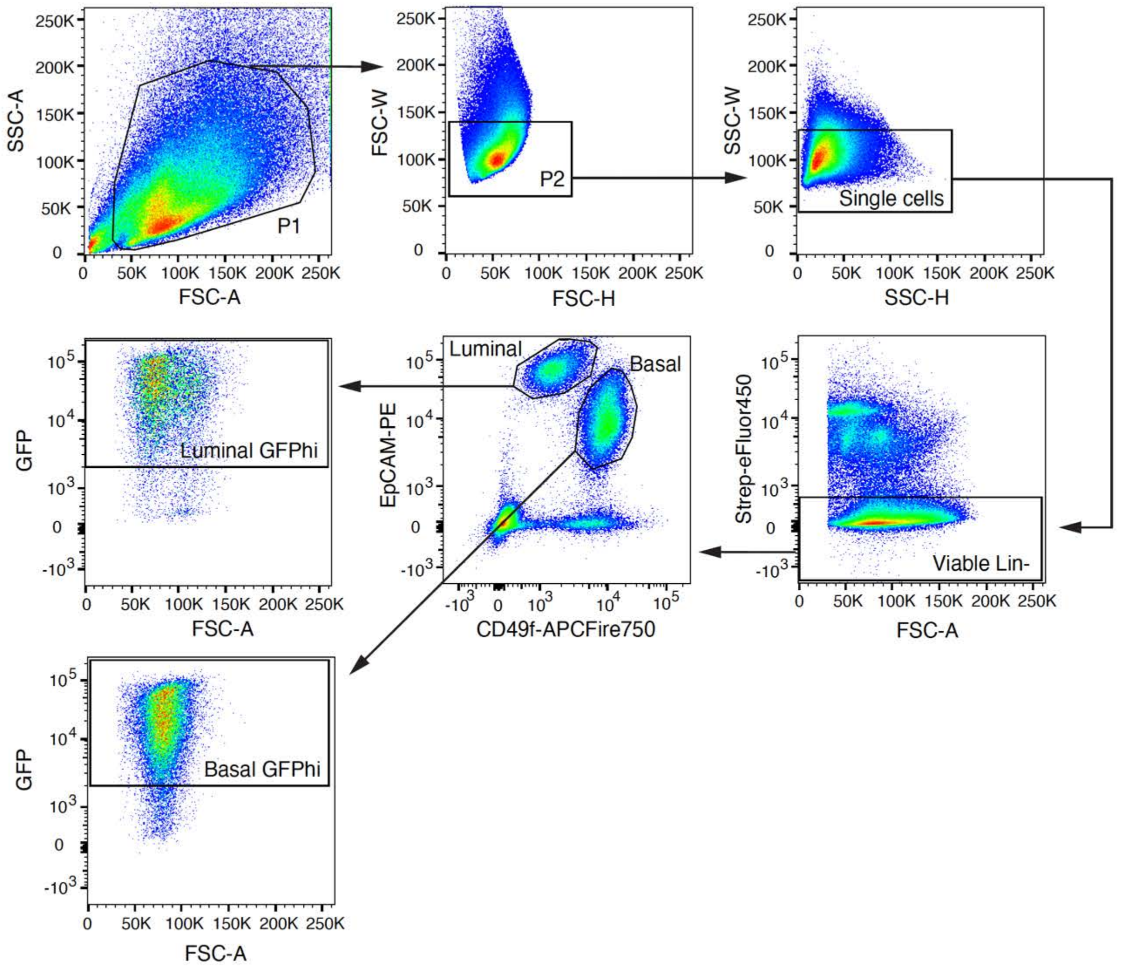


**Additional Figure 4:** sorting strategy used in Figure 2c to isolate basal and luminal cells expressing shTrps1 (GFPhi) from ubiquitous shTrps1 model mice.

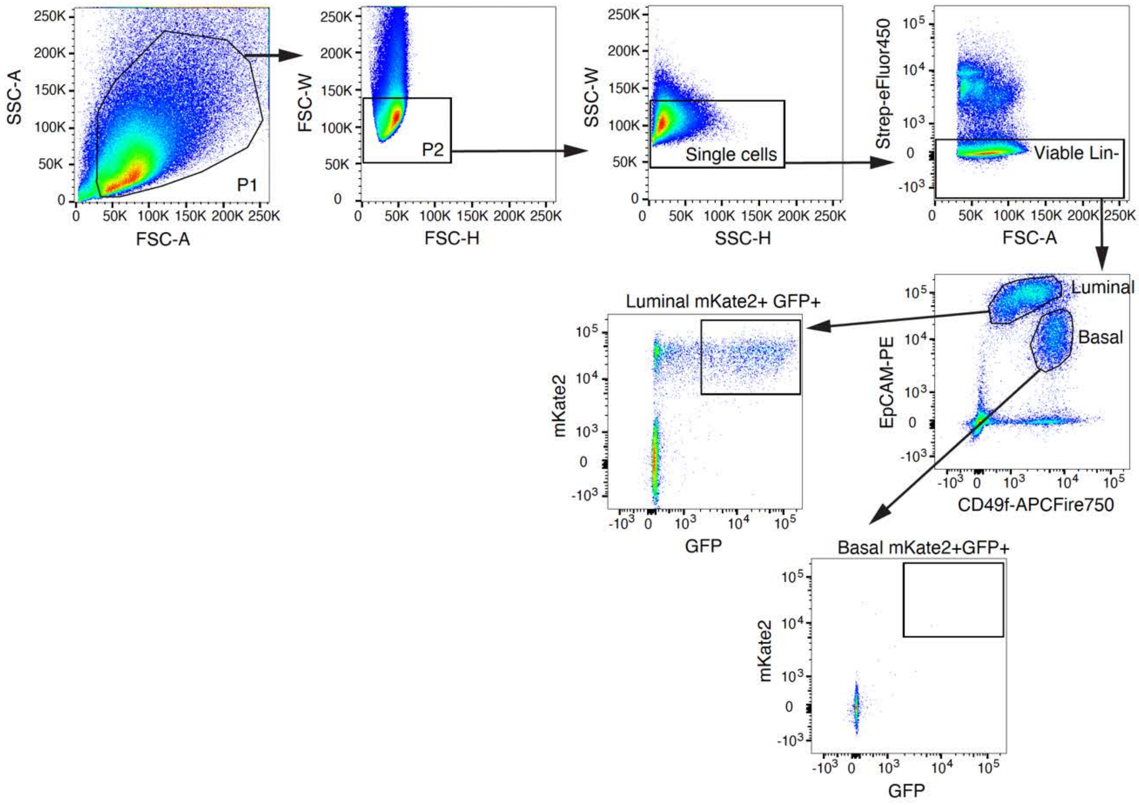


**Additional Figure 5**: sorting strategy used in Figure 2I to isolate mammary basal and luminal cells expressing shTrps1 (mKate2+ GFP+) from luminal-specific shTrps1 model mice.

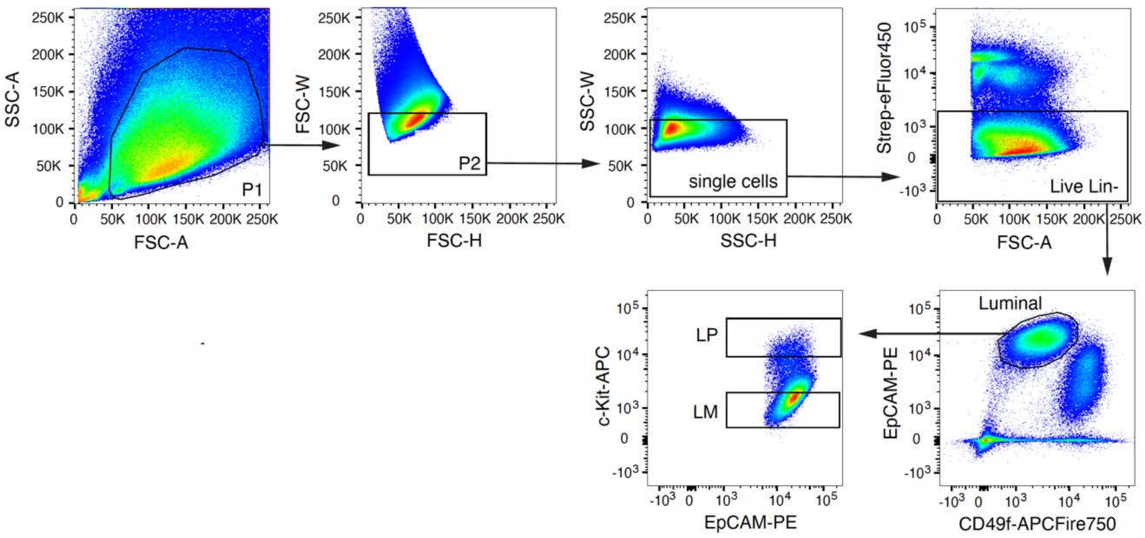


**Additional Figure 6**: sorting strategy used in Figure 3 to isolate mammary progenitor and mature luminal cells from wild type mice.

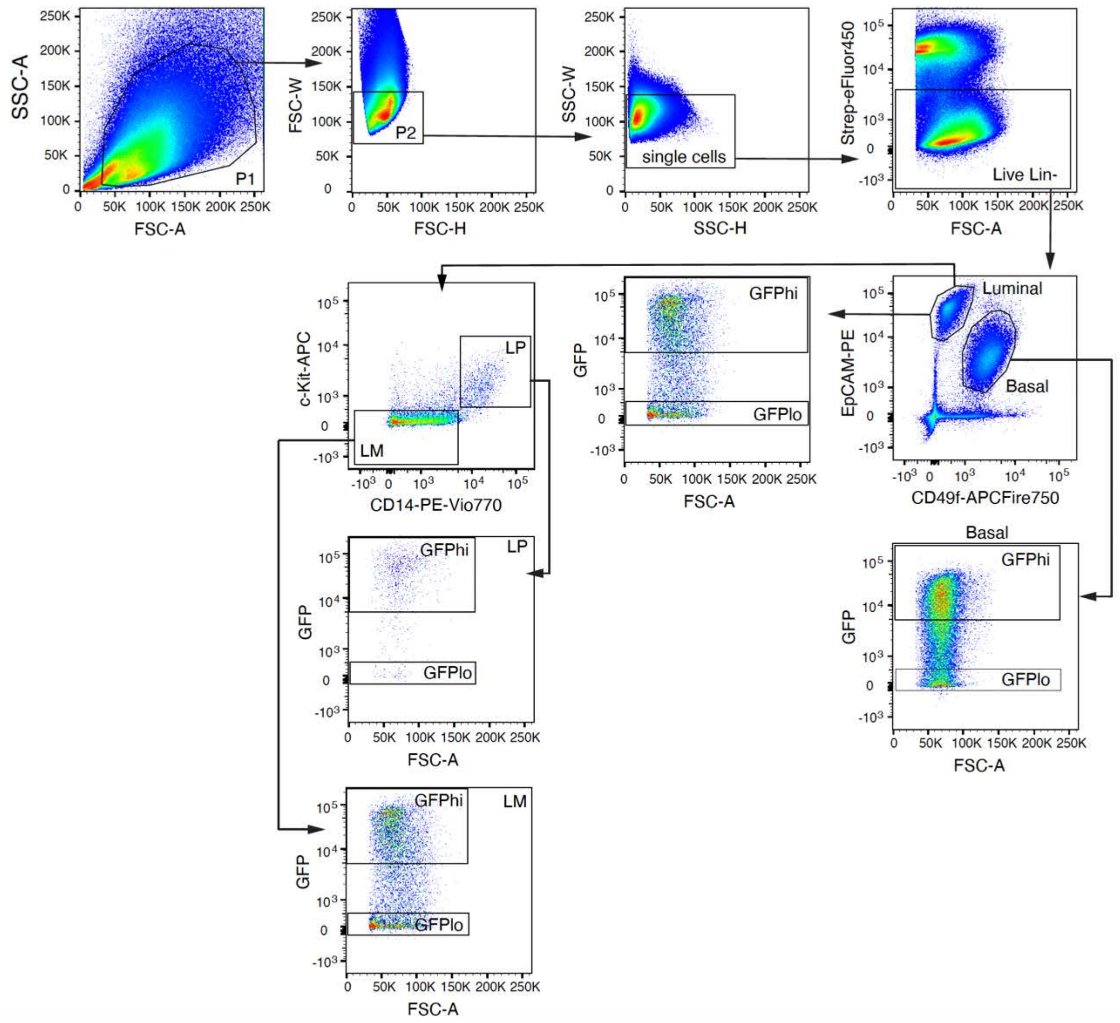


**Additional Figure 7**: gating strategy used in Figure 4a to analyze the proportion of GFPhi and GFPlo cells in the different mammary epithelial subpopulations: Basal, Luminal, Luminal mature (LM) and Luminal progenitor (LP).

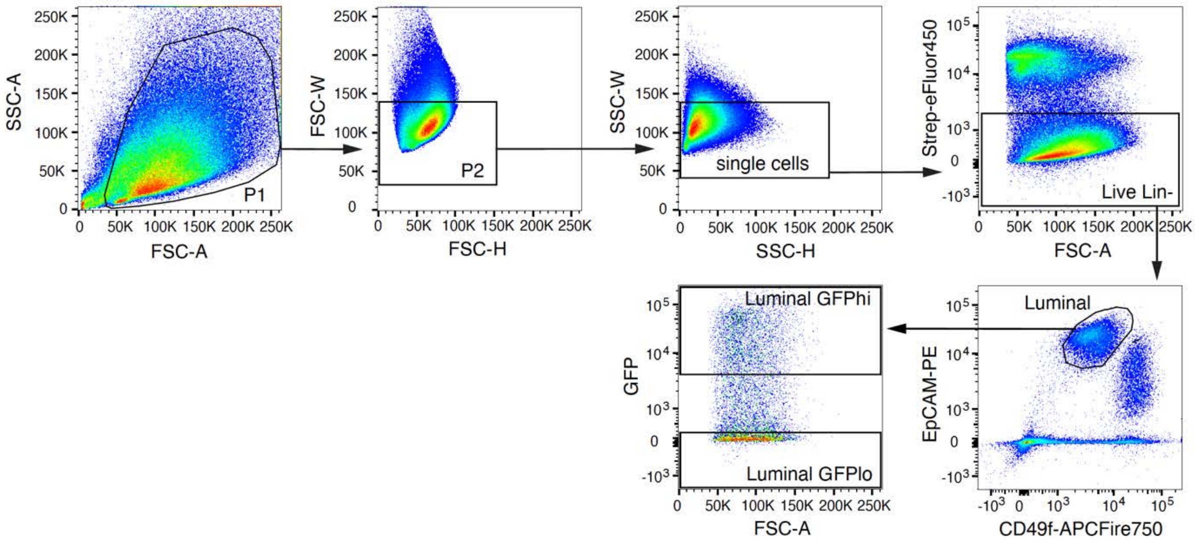


**Additional Figure 8**: sorting strategy used in Figure 4d-e to isolate mammary luminal cells expressing high shTrps1 (GFPhi) or expressing low of no shTrps1 (GFPlo) for a colony forming assay.

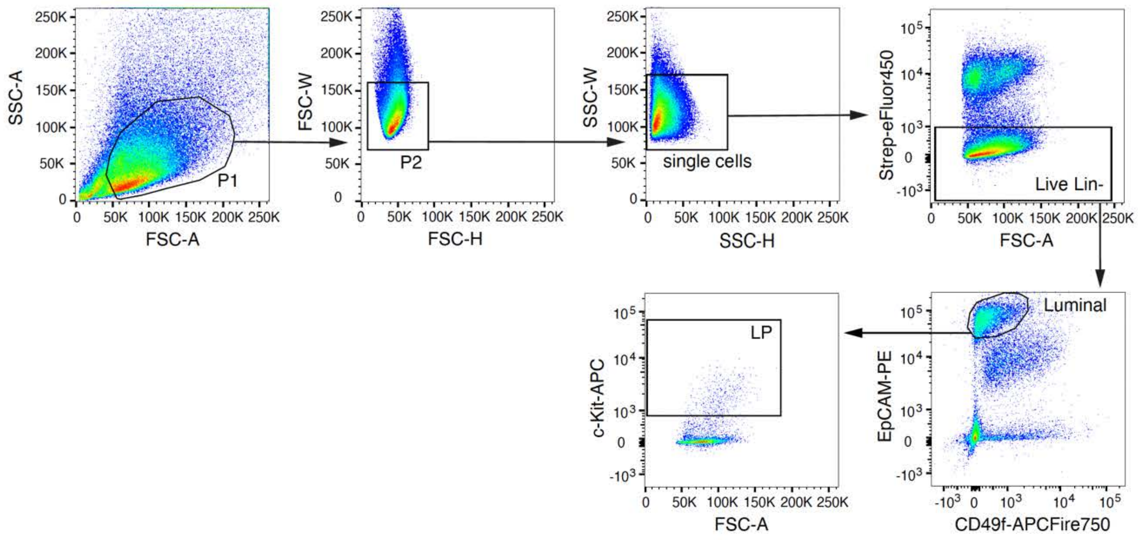


**Additional Figure 9**: sorting strategy used in Figure 5o-p to isolate mammary luminal progenitor cells from wild type mice for colony forming assay.

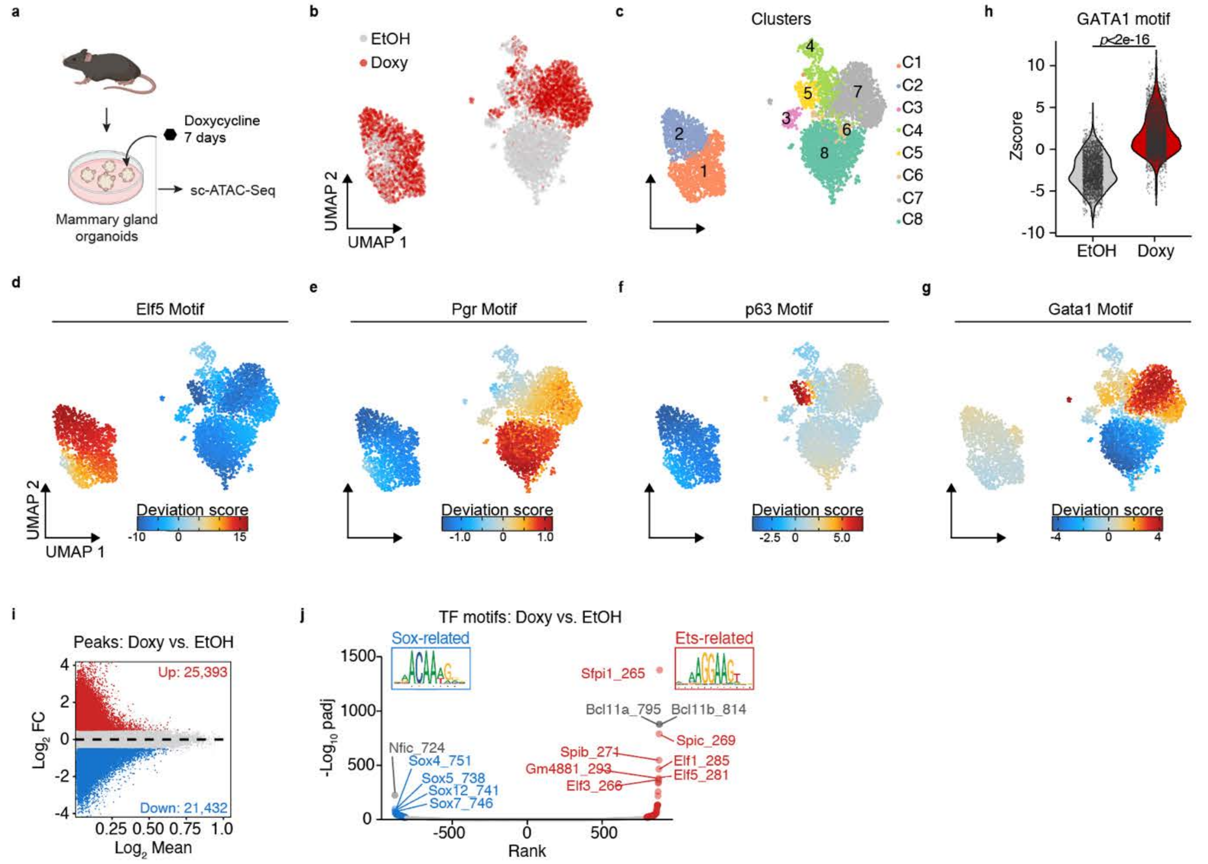


**Additional Figure 10: TRPS1 prevents LP differentiation**

(a) Experimental design: organoids prepared from mammary glands isolated from wt BL6 mice were treated with doxycycline for 7 days and subjected to sc-ATAC-Seq

(b)-(c) UMAP dimensionality reduction plots of the mammary organoid cells. (b) Cells treated with Dox or Ethanol are highlighted in red and grey respectively, (c) Graph-based clusters.

(d)-(g) UMAP dimensionality reduction plots of the mammary organoid cells showing the motif accessibility for the indicated TF.

(h) GATA1 motif enrichment in sc-ATAC-Seq peaks from Dox vs Ethanol-treated cells. Wilcoxon rank-sum test.

(i) Volcano plot of the differential sc-ATAC-Seq peaks from Dox vs Ethanol-treated cells.

(j) TF Motif enrichment in the up (red) or down (blue) regulated ATAC-Seq peaks in Dox vs Ethanol-treated cells.

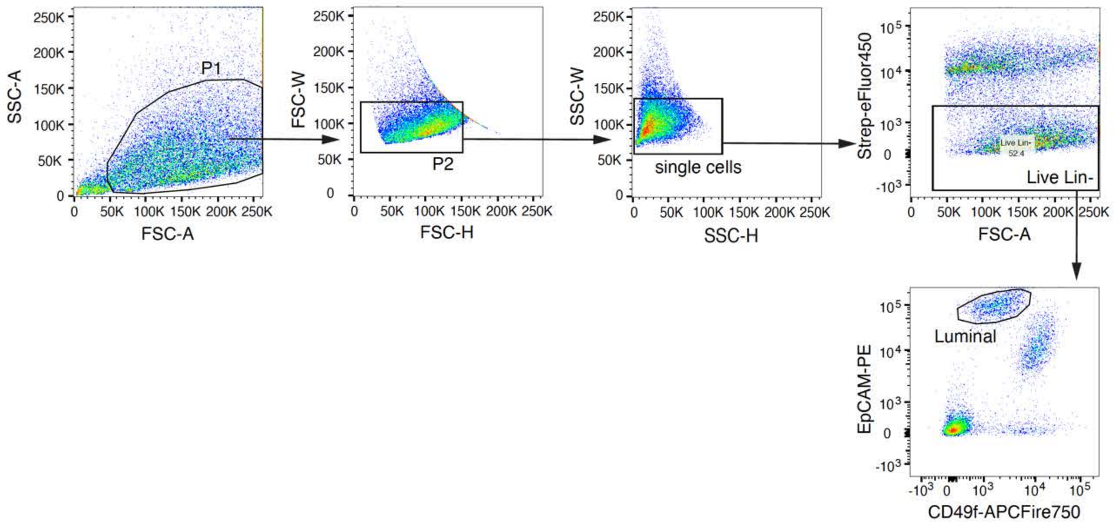


**Additional Figure 11**: sorting strategy used in Figure 6a to isolate mammary luminal cells from wild type mice for CUT and RUN.

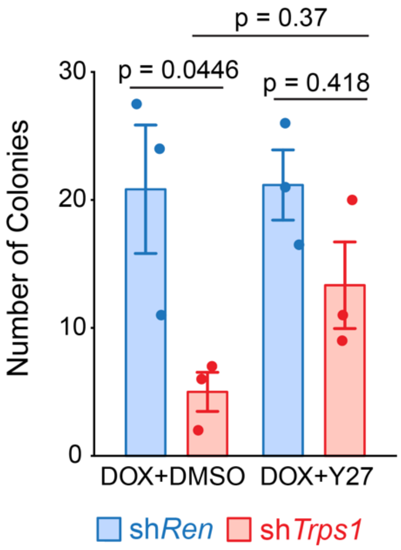


**Additional Figure 12**:
Number of colonies formed by mammary luminal progenitors sorted from shTrps1 and shRen control animals and treated *in vitro* with doxycycline (DOX) or a combination of doxycycline and the Y-27632 ROCK inhibitor (Y27).
One-way ANOVA with Tukey HSD post hoc test. Indicated *p* values are *p* adjusted.
